# Radical Mastectomy Efficiently Improves Long-Term Clinical Outcomes in Dogs with Malignant Mammary Tumors

**DOI:** 10.3390/ani14243687

**Published:** 2024-12-20

**Authors:** Seung-Hyun Kim, Dae Sung Yoo, Chul-Ho Park, Sang-Ho Lee, Ju-Hwan Lee, Taeho Ahn, Bock-Gie Jung, Jun-Gyu Park, Sang-Ik Park, Chun-Sik Bae

**Affiliations:** 1 Department of Veterinary Surgery, College of Veterinary Medicine, Chonnam National University, Gwangju 61186, Republic of Korea; trazet08@gmail.com; 2 College of Veterinary Medicine, Chonnam National University, Gwangju 61186, Republic of Korea; shanuar@jnu.ac.kr (D.S.Y.); cnu06806@jnu.ac.kr (J.-H.L.); thahn@jnu.ac.kr (T.A.); 3 Suncheon Bay Animal Hospital, Suncheon-si 58016, Republic of Korea; schbay@naver.com; 4 4rest Animal Medical Center, Jeonju-si 54828, Republic of Korea; 4restamc@naver.com; 5 Department of Veterinary Microbiology, College of Veterinary Medicine, Chonnam National University, Gwangju 61186, Republic of Korea; bjung@jnu.ac.kr; 6 Department of Veterinary Zoonotic Diseases, College of Veterinary Medicine, Chonnam National University, Gwangju 61186, Republic of Korea; kingsalt@jnu.ac.kr; 7 Department of Veterinary Pathology, College of Veterinary Medicine and BK21 FOUR Program, Chonnam National University, Gwangju 61186, Republic of Korea

**Keywords:** canine mammary gland tumors, complete excision, radical mastectomy, survival time, disease-free interval

## Abstract

The incidence of mammary gland tumors in dogs is a significant issue, characterized by notable rates of malignancy, recurrence, and disease burden. However, the lack of comprehensive information and correlated studies has presented challenges in establishing an effective surgical treatment strategy. In this study, a cohort of 95 dogs diagnosed with mammary tumors underwent evaluation using various surgical methods, medications, ovariohysterectomy, and assessments of margin completeness to analyze clinical outcomes. Significant clinical outcomes were observed following radical mastectomy and conservative surgeries, irrespective of TNM stages and histopathological grade of malignancy. Complete excision demonstrated significantly improved survival time and disease-free interval compared to incomplete excision. It is noted that complete surgical excision provides benefits, even in cases where lymphatic invasion is present. However, further investigations are necessary to validate the clinical results and mitigate side effects from radical mastectomy. Despite postoperative complications, several studies continue to advocate for more radical mastectomies due to their favorable risk-benefit ratio. Moreover, advanced inpatient treatments can now effectively manage or mitigate many of these complications. This study emphasizes the critical role of surgical proficiency and postoperative medication in treating malignant mammary tumors in dogs, and it provides insights that may contribute to the development of more effective treatment strategies for this condition.

## Simple Summary

In this study, a cohort of 95 dogs diagnosed with mammary tumors underwent evaluation through multiple surgical techniques, alongside medication and ovariohysterectomy. The assessment included margin completeness to analyze clinical outcomes. Complete excision of the tumors has been shown to enhance survival time, extend disease-free intervals, and improve the overall well-being of the animals across different clinical stages. The findings suggest that complete surgical excision provides benefits, even in cases where lymphatic invasion is present. Radical mastectomy, which may disrupt potential lymphatic routes for tumor invasion and recurrence, demonstrated better clinical outcomes compared to conservative therapy. However, further investigations are necessary to validate these clinical results and address the side effects associated with radical mastectomy. Despite the possibility of postoperative complications, studies continue to advocate for more radical mastectomies due to their favorable risk-benefit ratio. Additionally, advancements in postoperative treatment can now effectively mitigate many of these complications. This study emphasizes the importance of radical mastectomy in treating malignant mammary tumors in dogs, providing insights that could lead to the development of more effective treatment strategies.

## 1. Introduction

The prevalence of mammary tumors in intact female dogs is notable, with around 40 to 50% of these tumors being histologically malignant [1]. Despite the known clinical and histological characteristics of mammary cancers, their variable biological behaviors pose a significant challenge in accurately estimating individual clinical outcomes based solely on these traits [2]. 

The prognosis of mammary tumors in dogs can be predicted by various solid factors, such as tumor size, ulceration, margin status, fixation to underlying structures, lymph node status, and stage [3]. Research has shown that dogs with tumor diameters larger than 3 cm have a significantly worse outcome compared to those with smaller tumors [4,5]. This is further supported by evidence of histological progression from benign to malignant with increasing tumor size [5]. Additionally, ulceration has been identified as an independent predictor of poor prognosis [3], and fixation to underlying structures has been associated with a significant reduction in the duration of the metastasis-free interval [6]. Furthermore, the completeness of surgical margins is a strong prognostic factor, as clean margins result in a better outcome [3,7].

Metastasis in mammary cancer in dogs primarily occurs through the lymphatic system [8]. As a result, consideration of the lymphatic route has been integral in clinical decision-making regarding the appropriate surgical approach for mammary tumors in dogs. Mastectomy primarily aims to extract tumor tissues and adjacent affected tissues, including lymphatics and lymph nodes. According to many studies, breaking down the potential route of tumor invasion and metastasis can improve clinical outcomes [3]. Therefore, in dogs with malignant mammary tumors, the choice of a surgical approach based on lymphatic drainage is crucial and may influence treatment outcomes.

The presence of lymph nodes and distant metastases at the time of diagnosis has also been associated with shorter disease-free interval (DFI) and overall survival (OS) times compared to survival times in dogs without metastases. In the context of adjuvant chemotherapy, it is routinely used in women with invasive breast cancer and has been shown in some circumstances to improve survival in women with this disease [9]. In dogs, there have been case reports of measurable tumor responses to paclitaxel, doxorubicin, carboplatin, and mitoxantrone [10,11]. It is still uncertain whether adjuvant chemotherapy improves the survival times of dogs treated with mammary gland carcinomas post-operatively. Interestingly, chemotherapy consisting of carboplatin and cyclooxygenase inhibitors could benefit OS [12]. More research is required to evaluate its controversial effect on dogs [13,14]. 

Surgical excision is the preferred treatment for most mammary carcinomas. In human oncology, radical mastectomy is prioritized for managing aggressive breast cancers, such as inflammatory breast cancer, to achieve optimal control of any residual disease [15]. However, in cases where radical mastectomy is not possible, modifications to the surgical approach may be necessary to reduce the extent of the tissue removed. Recent studies have highlighted the survival benefits of macroscopically complete surgical excision [16]. Patients who have no visible residual disease after surgery experience significantly longer median overall survival compared to those who have any residual disease.

Breast-conserving surgery (BCS) is a commonly used approach in the treatment of human breast cancer, aiming to improve patient quality of life and address psychological concerns [17]. However, BCS may not be suitable for all patients, especially young women with certain risk factors, such as breast cancer with ductal carcinoma in situ (DCIS), tumor with lobular histology, a family history of genetic disorders, and carrying BRCA 1/2 mutations [18]. For these patients, conservative surgery is not recommended. Additionally, research has consistently shown that patients with positive margins following surgery have a higher risk of local recurrence compared to those with negative margins [19]. This underscores the importance of complete excision and radical surgical techniques.

This retrospective study aims to identify prognostic factors associated with survival in a specific group of dogs and compare survival data for dogs treated with incomplete versus complete excision. The study seeks to analyze the impact of radical mastectomy on survival rates and perioperative morbidity in patients with malignant mammary tumors compared to a conservative surgical approach.

## 2. Materials and Methods

### 2.1. Recruitment of Subjects and Interview

A total of 95 female or spayed female dogs exhibiting spontaneous mammary gland tumors (MMTs) were selected for surgical treatment and participation in a 3-year post-operative follow-up study with the consent of their owners. The study protocol adhered to the guidelines of the Chonnam National University Animal Hospital for the ethical treatment of animals used for scientific research and was approved by the Institutional Animal Care and Use Committee (IACUC) of Chonnam National University.

Data pertaining to the animals, including weight, age, reproductive status, and prior use of medications for estrus control, were collected and recorded. Each animal underwent clinical staging, which involved a comprehensive physical examination, three-view thoracic radiographs, and a complete abdominal ultrasound evaluation. Subsequently, the owners were interviewed via telephone to obtain information about their dog’s diet one year prior to diagnosis, body conformation according to the canine body condition score (BCS) system, medical and reproductive history, management practices, and survival post-diagnosis.

### 2.2. Surgical and Non-Surgical Treatment

The inclusion criteria for this review were studies that compared outcomes in female dogs with mammary neoplasia following different surgical interventions or reported outcomes following a single surgical intervention without a control group. Surgical interventions were categorized based on their extent, including lumpectomy, simple mastectomy, regional mastectomy, and radical mastectomy. These interventions were defined as follows [16]: Lumpectomy involved the removal of the tumor only; simple mastectomy involved the removal of the affected gland only; regional mastectomy involved the removal of the affected gland and glands that shared lymphatic drainage. Given the five pairs of mammary glands from cranial to caudal parts of the body, the first, second, and third mammary glands share the same lymphatic route to the axial lymph node. In contrast, the third, fourth, and fifth mammary glands share the same lymphatic route to the superficial inguinal lymph node [20]. Along with the associated lymph nodes, radical mastectomy was performed to achieve the complete removal of the mammary chain and related lymph nodes, either unilaterally or bilaterally.

In addition to surgical options, palliative treatment is available for dogs, which involves administering drug medications, such as NSAIDs, without any surgical procedures.

### 2.3. Histopathological Examination and Staging

The removed tumors were fixed in 10% neutral buffered formalin for 48 h, measured in their largest diameter, and then categorized as <3 cm or ≥3 cm for statistical analysis. Tumors <1 cm were paraffin-embedded in a single block, while larger tumors were sequentially cut at 5 mm intervals to provide representative tissue blocks of the entire lesion. Following dehydration and embedding in paraffin wax, 3 μm sections were obtained from each block. These sections were stained using hematoxylin and eosin (HE), and histological classification was performed by two pathologists (FG and IA) using the criteria from the World Health Organization for the histological classification of mammary tumors in domestic animals [1]. For statistical purposes, tumors were grouped as complex carcinomas, simple carcinomas (solid, tubulopapillary, micropapillary, and anaplastic), and other (inflammatory carcinoma, osteosarcoma, mucinous carcinomas, spindle cell carcinomas, carcinosarcomas, and carcinomas in benign tumors).

The mitotic index was calculated and classified as low (fewer than 10 mitotic figures per 10 high-power fields), moderate (10 to 19 per 10 high-power fields), and high (more than 20 per 10 high-power fields). Histological grading was determined according to the Nottingham method as Grade I (well-differentiated), Grade II (moderately differentiated), and Grade III (poorly differentiated), as previously described in canine mammary tumors [21]. Tumor growth was classified as expansive (cohesive and well-delimited mass pushing normal surrounding tissues) or invasive (infiltrative growth or lymphatic or blood vessel invasion) [1]. The presence of necrosis and squamous metaplasia, when detected, was also recorded.

### 2.4. Statistical Analysis

Survival analysis was conducted using the Kaplan–Meier method, and differences between the groups for each variable were assessed using the log-rank test [1]. In the overall survival (OS) study, dogs were censored if they died from causes unrelated to MMTs or were lost to follow-up. In the disease-free survival (DFS) study, dogs were censored if they were lost to follow-up or died from causes unrelated to MMTs before developing recurrences or metastases. Cox regression analysis was employed to estimate the hazard of recurrence or distant metastasis and the hazard of tumor-related death for each variable [22]. Variables significantly associated with OS or DFS in univariate analyses were included in the multivariable Cox proportional hazards model using the forward stepwise method to select outcome predictors that retained significance while controlling for confounding variables. Due to a high number of missing cases, RLN status was not considered for inclusion in the multivariable model. The significance level was set at *p* < 0.05. Statistical analysis was performed using PASW Statistics 18.0 and GraphPad Prism (version 9.4.2, GraphPad Software Inc., San Diego, CA, USA). Data were expressed as mean  ±  standard deviation (SD), and statistical differences were determined using the Student’s *t*-test, χ^2^ test, and one-way or two-way ANOVA. Statistical significance was denoted as follows: * for *p*  <  0.05, ** for *p*  <  0.01, *** for *p*  <  0.001, and **** for *p*  <  0.0001.

## 3. Results

### 3.1. Characteristics of Patient Factors

The study identified records for 118 dogs with mammary carcinoma who underwent surgical treatment. Fifteen dogs were excluded due to insufficient information, and an additional eight dogs were lost to follow-up after surgery within 22 days, excluding 23 cases. This left a final population of 95 dogs for analysis. Information on various parameters, including age, weight, breed, stage, neuter status, tumor size, number of tumors, and histopathological stage, was available for all included dogs (Table 1). However, information regarding lymphatic invasion, ulceration, and surgical margins was unavailable for all cases. 

The study also evaluated tumor necrosis, ulceration, and surgical margins findings. Additionally, the treatment modalities after surgery were detailed, with a significant number of dogs receiving a combination of NSAIDs and/or piroxicam following surgery.

Thoracic radiographs were performed on 72 dogs, and abdominal ultrasonography was conducted on 22 dogs. The median age of the affected dogs was 10 years, with a range of 5–18 years (Table 2). The median body weight was 5.8 kg, ranging from 3 kg to 32 kg. The majority of the population consisted of intact females, totaling 76 dogs, with a smaller subset of 19 neutered dogs. Tumor size varied, with T1, T2, and T3 tumors observed in 25, 38, and 32 dogs, respectively. Metastasis at the time of presentation was found in five dogs, with various patterns of metastases identified. These five dogs were excluded from surgery and instead received conservative therapy.

Almost half of the dogs had multiple tumors, with 45 out of 95 dogs affected. According to the modified clinical staging system used, the distribution of dogs across different stages was as follows: 28 in stage 1, 16 in stage 2, 28 in stage 3, 15 in stage 4, and 8 in stage 5. Lymphatic invasion was identified in 24.0% of the cases where information was available.

The study found that 35% (35 out of 95 dogs) of the dogs had histologic presence of tumor necrosis, while the tumor was ulcerated in 22% (21 out of 95 dogs) of the cases (Table 3). Surgical margins were considered histologically incomplete in 19% (13 out of 69 dogs) of the dogs and complete in 81% (56 out of 69 dogs). Regarding treatment, 9% (9 out of 95 dogs) of the dogs did not receive treatment, 18% (17 out of 95 dogs) were treated with NSAIDs and/or piroxicam without surgery, 73% (69 out of 95 dogs) received surgery, including lumpectomy (LM), partial mastectomy (PM), unilateral mastectomy (UM), and bilateral mastectomy (BM), and 60% had ovarian hysterectomy (OHE) along with surgical treatment. All surgeries were completed in one procedure, regardless of the surgical type.

### 3.2. Characteristics of Treatment and Clinical Outcomes

In a cohort of 95 dogs, to acquire information during our scheduled follow-up assessments, we engaged directly with pet owners to ascertain the condition of their dogs. When pet owners were unavailable for contact, we initiated follow-up phone calls to verify the animals’ health status and confirm any reported deaths. 

The outcomes were as follows: 25 dogs (26.3%) were confirmed deceased, with 17 of these deaths attributed to mammary carcinoma, 4 due to causes not related to mammary tumors, and 4 cases where the cause of death remained unknown. Additionally, 46 dogs (48.4%) were lost to follow-up, with a median follow-up duration of 878 days and a range of 32 to 2154 days, four of which were lost after 200 days. Finally, 24 dogs (25.3%) were reported to still be alive, with a median follow-up duration of 879 days and a range of 27 to 1896 days (Table 3).

In addition, postoperative complications were investigated. Of the 57 dogs that underwent OHE, none exhibited estradiol-related complications, such as urinary incontinence. Severe pain requiring hospitalization was noted in one out of five UM cases and three out of nine BM cases, with an average hospital stay of approximately 3.5 days. Secondary infection or inflammation at the surgical site occurred in 12 out of 95 cases, and all affected dogs were cured with disinfection and non-invasive medical treatment, leaving no sequela. Surgical site dehiscence requiring re-suturing occurred in about three out of ninety-five cases. Preoperative tests were conducted to assess the feasibility of anesthesia and surgery, and as a result, no preoperative deaths, including table deaths, occurred.

### 3.3. Prognostic Factors

Upon univariate analysis, several factors were found to be significantly associated with survival, including age (*p* = 0.031), clinical stage (*p* < 0.024), presence of lymphatic invasion (*p* < 0.003), metastases at diagnosis (*p* = 0.006), and incomplete surgical margins (*p* < 0.012) (Table 4). These factors were subsequently subjected to multivariate analysis. The results of the multivariate analysis revealed that only increasing clinical stage (*p* = 0.037), presence of lymphatic invasion (*p* = 0.009), and incomplete surgical margins (*p* = 0.018) were independent predictors of poor survival. The remaining factors examined were not deemed to be statistically significant.

We produced the Kaplan–Meier survival curve to compare the median survival rates between untreated and treated dogs. Throughout the investigation, we prioritized surgical treatment over medication when the dog was deemed fit for surgery, and this approach was consistently recommended to all dog owners from the start. However, the choice among available treatment options ultimately depends on the owner’s willingness to proceed.

**Figure 1:**
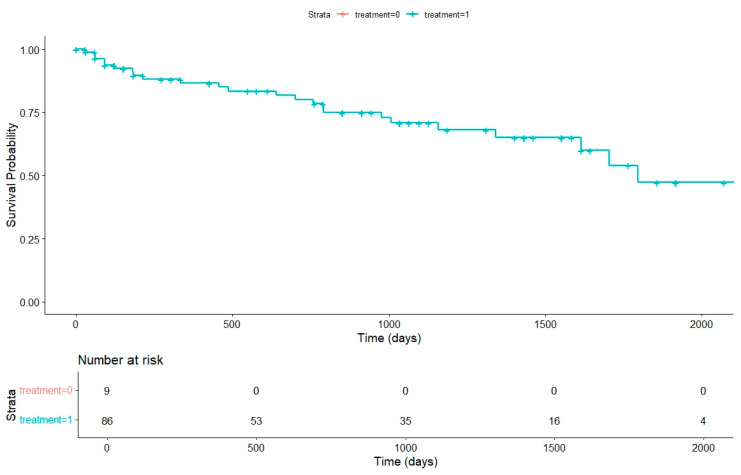
Kaplan–Meier survival curve comparing the effect on survival for nine dogs with no treatment (pink line, which is covered by the X and Y axes due to no survival rates reported for this group) entirely censored to eighty-six dogs with surgical treatments and/or medication (blue line; median survival: 775 days). *p* = 0.005.

Out of ninety-five dogs, nine were not treated, while eighty-six received treatment, which included palliative care (seventeen dogs), incomplete excision (thirteen dogs), and complete excision (fifty-six dogs). The median survival for the untreated group could not be determined because all dogs in that group were censored due to their short survival times. In contrast, the median survival for the treatment group was 775 days, indicating that treatment contributes to improved survival outcomes (Figure 1).

The independent prognostic significance of histologic margins was observed overall (*p* < 0.001), among the 64 dogs with available histologic margin assessments. Nine dogs had no treatment, and none were censored, with a median survival of 172 days (95% CI: 73–469) (Table 5). Furthermore, seventeen dogs received NSAIDs without surgical treatment, and two were censored, with a median survival of 140 days (95% CI: 160–235). Thirteen dogs had incomplete excision, and one was censored, with a median survival of 324 days (95% CI: 261–756). Finally, 56 dogs received complete excision, and 22 were censored, with a median survival of 992 days (95% CI: 934–1217), highlighting the importance of complete resection in cancer therapy.

**Figure 2:**
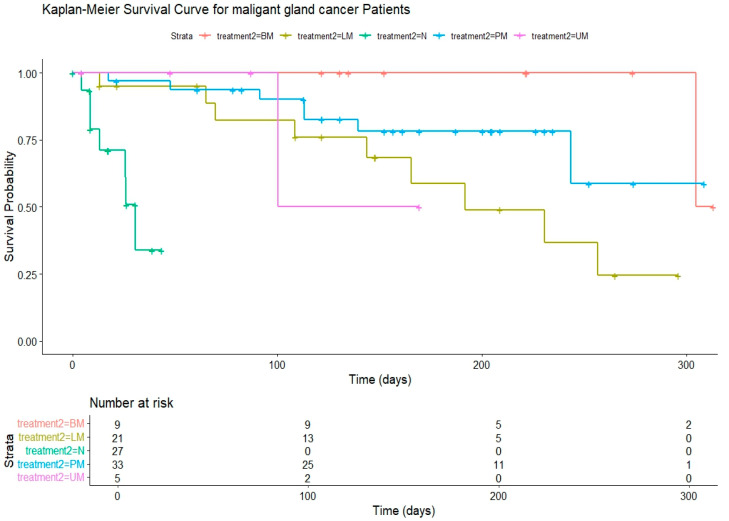
Kaplan–Meier survival curve comparing the effect on survival for surgical types. Abbreviations: N, no treatment; N/A, not applicable; LM, lumpectomy; PM, partial mastectomy; UM, unilateral mastectomy; BM, bilateral mastectomy.

The findings above indicate that complete excision leads to more favorable outcomes, resulting in a significant reduction in morbidity and mortality compared to incomplete excision. Specifically, among the 56 dogs that underwent complete excision, subgroups were identified based on the type of procedure: lumpectomy (LM), partial mastectomy (PM), unilateral mastectomy (UM), and bilateral mastectomy (BM). The choice of surgery is determined based on specific criteria, such as tumor size and location, potential extension to regional lymph nodes, tumor adherence or fixation to surrounding tissues, and the total number of lesions [24]. Among this cohort, 21 dogs underwent LM, with a median survival rate of 879 days (95% CI: 625–1150). Additionally, five dogs underwent UM, with a median survival rate of 587 days (95% CI: 187–927), while thirty-three dogs underwent PM, exhibiting a median survival rate of 966 days (95% CI: 795–1150). Notably, nine dogs underwent BM, with a median survival rate of 1425 days (95% CI: 1123–1830), highlighting the importance of radical mastectomy in tumor resection. In addition, significant survival probabilities were observed between treatment groups, where BM exhibited the best survival probability among the dogs that received surgical treatments (Figure 2).

To assess the impact of positive and negative margins on survival time, complete excision was compared to incomplete excision across each clinical stage (Table 6). As anticipated, complete excision consistently demonstrated superior clinical outcomes compared to incomplete excision across all stages (Table 6).

To assess the impact of OHE on the clinical outcomes depending on surgery types, we conducted an additional Cox hazard regression analysis, including OHE as an explanatory variable. As a result, our analysis did not identify a significant association between survival time and OHE in the data from our study (Table 7).

## 4. Discussion

The increasing prevalence of malignant mammary gland tumors (MGT) in dogs, influenced by environmental factors and extended life expectancy, poses a significant concern. Over 50% of these cases are diagnosed as malignant, leading to economic burdens and treatment limitations for both pet owners and veterinary clinicians [25]. MGT is also notorious for its high rates of recurrence and metastasis [26]. Timely diagnosis and appropriate surgical intervention for malignant mammary carcinoma are, therefore, crucial for the overall well-being of companion animals. This study aimed to evaluate surgical treatments categorized as incomplete and complete excision, as well as palliative treatment, for dogs with malignant mammary tumors. Surgical methods were assessed based on prognosis, and clinical outcomes, including DFI and OS, were evaluated in a cohort of 95 dogs over a long-term period.

Previous studies have well-documented lymphatic invasion’s impact on clinical outcomes, underscoring its role as a negative prognostic factor [27,28]. Our study confirms that lymphatic invasion independently predicts reduced survival time and higher rates of recurrence. Furthermore, ulceration has been recognized as a negative prognostic indicator, and our investigation also identifies it as an independent predictor of poor prognosis within this specific canine population [3]. 

Of particular note is the critical role of surgical margin completeness in dogs with non-metastasized tumors (stages 1–3) and those exhibiting histologic evidence of lymphatic invasion. This underscores the potential existence of an early ‘locoregional’ phase of lymphatic invasion [29], especially in breast cancer, suggesting that affected patients may benefit from wide excision. Analogous to findings in human breast cancer patients, where improved local cancer control at 5 years has been shown to enhance overall 15-year survival, our data imply that aggressive local surgery to achieve complete margins may similarly improve overall survival in dogs with mammary cancer, even in the presence of histologically described lymphatic vessel invasion [30].

In our investigation into the positive impact of radical mastectomy on OS and DFI, we conducted an evaluation of various surgical methods, including LM, PM, UM, and BM. Our findings revealed that BM consistently yielded the most favorable outcomes over time, underscoring the potential for radical mastectomy to enhance both DFI and OS significantly. Furthermore, our analysis indicated that complete excision with a clear negative margin of the tumor consistently resulted in superior outcomes compared to incomplete excision, irrespective of the specific surgical method employed.

Importantly, our study highlighted that complete excision consistently enhances OS and DFI across all clinical stages, suggesting its preferential efficacy in all cases. Additionally, several studies have reported that the mean survival time for dogs with early-stage TNM classification was 22.2 ± 0.8 months, while for those with advanced TNM classification, it was 11.2 ± 3.6 months [31]. Furthermore, the 2-year survival rate for dogs with benign tumors was 90.2%, whereas for malignant tumors, it stood at 67.3%, exhibiting a statistically significant survival rate between benign and malignant tumors. Accordingly, our investigation demonstrated a notable disparity in survival outcomes following radical mastectomy, particularly in cases of mammary carcinoma across different TNM stages. Additionally, a more favorable survival time was found compared to previous reports, emphasizing the critical importance of surgical proficiency and following medication in the treatment of canine mammary gland tumors.

In the field of human oncology, BCS is a widely utilized approach for the treatment of breast cancer, with the primary goal of enhancing patient quality of life and addressing psychological concerns [17]. However, conservative surgery is limitedly recommended to patients because it could be contraindicated, especially in young women with specific risk factors such as breast cancer with DCIS, tumors with lobular histology, a family history of genetic disorders, and carrying BRCA1/2 mutations [18]. Additionally, research has consistently demonstrated that patients with positive margins following surgery are at a higher risk of local recurrence compared to those with negative margins [19], highlighting the significance of complete excision and radical surgical techniques. Furthermore, in contrast to human medicine, adjunctive chemotherapy in dogs has shown limited effectiveness, with no demonstrated improvement in OS and DFI [3]. Statistical analysis did not reveal any significant benefit from chemotherapy overall or from the most commonly used drugs, such as carboplatin and doxorubicin, which aligns with the findings of a previous study [6].

A recent publication delineated the criteria deemed essential for enhancing the validity of studies to assess prognostic factors in dogs diagnosed with mammary carcinoma [32]. It was observed that patients with mammary carcinomas exhibiting higher histological grades tend to have shorter survival times, and the specific type of carcinoma appears to be correlated with prognosis [33]. Notably, simple carcinomas have been associated with a poorer prognosis compared to other carcinoma types [21]. However, due to the study’s retrospective nature, certain limitations were encountered, including the absence of standardized monitoring and staging. Tumor-specific survival and disease-free interval were posited as superior endpoints in this study; however, the reliability of obtaining this information was impeded by the considerable variation in the frequency of follow-up time. Furthermore, assessing individual response rates for each chemotherapy agent was challenging, given that most dogs received multiple chemotherapy agents as adjunctive therapy. Despite including a relatively large number of dogs in the study, the numbers dwindled when conducting a subset analysis, thereby diminishing the statistical power of the study.

In a recent publication, Matos (2012) outlined the essential criteria for improving the validity of studies assessing prognostic factors in dogs diagnosed with mammary carcinoma [32]. The study observed that dogs with mammary carcinomas of higher histological grades tend to have shorter survival times, and the specific type of carcinoma appears to be correlated with prognosis [33]. Particularly, simple carcinomas have been associated with a poorer prognosis compared to other carcinoma types [21]. However, the retrospective nature of the study posed certain limitations, including the absence of standardized monitoring and staging. Tumor-specific survival and DFI were posited as superior endpoints in this study; however, obtaining this information reliably was impeded by the considerable variation in the frequency of follow-up time. Despite the inclusion of a relatively large number of dogs in the study, the numbers dwindled during subset analysis, thereby diminishing the statistical power of the study. In addition, in this study, small breeds were included due to regional and national preferences for dog breeds, which limited the reflection of the entire canine population. Thus, meta-analysis with large data regarding clinical outcomes and more extensive studies on postoperative complications should be required. 

The study reaffirmed the significance of previously identified prognostic factors in predicting survival among this cohort of dogs. The findings also suggest that complete surgical excision confers benefits, even in cases where histological evidence of lymphatic invasion is present. A radical mastectomy, as opposed to a simple mastectomy, has been associated with more favorable outcomes, including achieving complete surgical margins, prolonged survival time, and an extended DFI. However, it is important to note that this approach is accompanied by certain drawbacks, such as prolonged hospitalization, severe pain necessitating opioid analgesics, and postoperative complications, including larger surgical wound size, longer procedure duration, surgical site infections, and an elevated postoperative mortality rate [34,35]. Notwithstanding these postoperative complications, several studies, including our own, continue to advocate for more radical mastectomies due to their favorable risk-benefit ratio. Moreover, many of these complications can now be effectively managed or mitigated with advanced inpatient treatments [35].

## 5. Conclusions

Our study highlighted that complete excision consistently enhances OS and DFI across all clinical stages, suggesting its preferential efficacy in all cases. It is noted that there is a significant disparity in survival outcomes following radical mastectomy, particularly in cases of mammary carcinoma across different TNM stages. Additionally, a more favorable survival time was found compared to previous reports, emphasizing the critical importance of surgical proficiency and subsequent medication in the treatment of canine mammary gland tumors.

The findings also suggest that complete surgical excision confers benefits, even in cases where histological evidence of lymphatic invasion is present. A radical mastectomy, as opposed to a simple mastectomy, has been associated with more favorable outcomes, including achieving complete surgical margins, prolonged survival time, and an extended DFI. Notwithstanding postoperative complications, several studies, including our own, continue to advocate for more radical mastectomies due to their favorable risk-benefit ratio. Moreover, many of these complications can now be effectively managed or mitigated with advanced inpatient treatments.

## Figures and Tables

**Table 1:** Modified TNM staging for the classification of mammary tumors from 95 dogs [23].

TNM Staging	N
T = tumor size	
T1 (tumor <3 cm maximum diameter)	25
T2 (tumor 3–5 cm maximum diameter)	38
T3 (tumor >5 cm maximum diameter)	32
N = reginal lymph nodes	
N_0_ (No evidence of lymph node metastasis)	57
N_1_ (Lymph node metastasis present)	18
M = distant metastasis	
M_0_ (no evidence of distant metastasis)	64
M_1_ (distant metastasis present)	8
TNM staging	
Stage 1	28
Stage 2	16
Stage 3	28
Stage 4	15
Stage 5	8

**Table 2:** Characteristics of 95 dogs with mammary tumors included in the study.

	N
Breed	
Maltese	25
Mixed	14
Shih Tzu	12
Poodles	11
Yorkshire Terrier	6
A. Cocker Spaniel	5
Pomeranian	4
Schnauzers	3
Jindo Dog	3
Other	8
Median age (years)	10 (5–18)
Median weight (kg)	5.8 (3–32)
Sex	
Female	76
Female neutered	19
Histopathological grade	
Benign	17
Grade I	10
Grade II	21
Grade III	16
Unidentified	31
Morphological diagnosis	
N/A	31
Carcinoma in situ	9
Tubulopapillary carcinoma	14
Complex carcinoma	19
Tubulopapillary carcinoma with bone and/or cartilage differentiation	6
Complex carcinoma with bone and/or cartilage differentiation	5
Solid carcinoma	5
Mammary gland osteosarcoma	2
Others (anaplastic carcinoma, inflammatory carcinoma, carcionosarcoma, spindle cell carcinoma, etc.)	4
Recurrence (within 3 years)	
Yes	14
No	81

**Table 3:** Characteristics of treatment and prognostic factors.

**Dog**	**Treatments**				**Monitoring**	**Clinical** **Staging**	**Recurrence (month)**	**ST**	**DFI**
	**Surgical** **Type**	**Additional** **Surgery**	**Medication**	**Effects**					
1	PM	OHE		IC	Death	G2	N	835	
2	LM	OHE		IC	LTFU	G4	1	164	28
3	NT			N	LTFU	G3	1	762	
4	LM	OHE		IC	Death	G1	N	1642	42
5	PM	OHE		CE	LTFU	G4	6	381	
6	LM			IC	Death	G4	2	324	192
7	PM	OHE		IC	Death	G4	N	627	67
8	PM			CE	LTFU	G1	UN	1425	
9	NT			N	LTFU	G4	24	524	
10	PM			CE	LTFU	G4	24	742	
11	NT			N	LTFU	G3	36	1854	
12	PM			IC	LTFU	G2	N	332	742
13	NT			N	LTFU	G3	6	581	
14	PM	OHE		IC	Death	G4	2	324	721
15	PM			CE	LTFU	G1	N	1107	1823
16	NT	OHE		P	LTFU	G1	N	343	
17	UM	OHE		IC	LTFU	G4	3	587	
18	UM	OHE		IC	Death	G1	N	1842	181
19	UM	OHE		IC	LTFU	G2	N	132	64
20	PM	OHE		CE	LTFU	G2	N	412	
21	NT		NS	P	LTFU	G1	N	2143	
22	PM	OHE		IC	LTFU	G3	N	2081	
23	UM	OHE		CE	LTFU	G2	N	1594	95
24	NT			N	LTFU	G1	N	1010	
25	LM			CE	LTFU	G1	N	974	
26	NT		NS	P	Death	G1	N	2028	
27	PM	OHE	NS	CE	LTFU	G4	N	878	
28	NT			N	LTFU	G3	N	1425	
29	BM	OHE	NS	CE	LTFU	G1	N	1581	
30	BM	OHE	NS	CE	Death	G2	N	748	
31	PM	OHE	NS	CE	LTFU	G3	5	472	
32	LM	OHE		CE	LTFU	G3	N	1041	
33	NT			N	LTFU	G3	N	1162	
34	LM	OHE		CE	Death	G2	N	1671	
35	NT		NS	P	LTFU	G1	N	1882	
36	NT		NS	P	LTFU	G1	N	1896	
37	LM	OHE		CE	LTFU	G2	N	1095	
38	NT			N	LTFU	G1	N	1438	
39	BM		NS	CE	LTFU	G4	N	133	
40	NT		NS	P	Death	G1	N	1031	
41	NT		NS	P	Death	G3	N	1303	
42	LM	OHE		CE	LTFU	G3	N	1752	
43	LM	OHE		CE	Death	G3	N	103	
44	PM	OHE		CE	LTFU	G1	N	1545	
45	LM			IC	Death	G3	N	468	
46	BM			CE	LTFU	G2	7	491	
47	UM	OHE		CE	LTFU	G1	N	457	
48	PM	OHE		CE	Death	G1	N	657	
49	BM		NS	CE	LTFU	G2	N	824	
50	BM		NS	CE	LTFU	G3	N	1390	
51	NT		NS	P	Death	G2	15	754	
52	PM		NS	CE	LTFU	G3	N	1026	
53	PM	OHE	NS	CE	LTFU	G1	36	1334	
54	PM	OHE		CE	Death	G1	N	1060	
55	LM	OHE		CE	LTFU	G1	N	577	
56	PM	OHE		CE	LTFU	G1	N	874	
57	PM		NS	CE	LTFU	G1	N	1184	
58	NT		NS	P	LTFU	G2	N	1211	
59	LM	OHE		CE	Death	G2	N	1087	
60	NT		NS	P	Death	G2	N	1091	
61	BM		NS	CE	LTFU	G1	N	788	
62	PM		NS	CE	LTFU	G3	N	966	
63	LM	OHE		CE	Death	G3	N	32	
64	NT	OHE	NS	P	LTFU	G3	N	124	
65	LM	OHE		CE	LTFU	G3	N	189	
66	PM	OHE	NS	CE	LTFU	G2	N	364	
67	PM	OHE	NS	CE	LTFU	G2	N	945	
68	LM	OHE		CE	LTFU	G1	N	846	
69	PM	OHE	NS	CE	LTFU	G3	N	918	
70	LM	OHE		CE	Death	G1	N	879	
71	PM	OHE		CE	LTFU	G1	N	879	
72	NT		NS	P	LTFU	G2	N	882	
73	PM	OHE		CE	LTFU	G2	N	835	
74	PM	OHE		CE	LTFU	G4	1	164	
75	LM	OHE		CE	Death	G3	1	762	
76	PM	OHE		CE	LTFU	G1	N	1642	
77	NT		NS	P	LTFU	G4	6	381	
78	PM	OHE	NS	CE	LTFU	G4	2	324	
79	NT			N	LTFU	G4	N	627	
80	PM	OHE		CE	LTFU	G1	UN	1425	
81	PM	OHE		CE	Death	G4	24	524	
82	PM	OHE		CE	Death	G4	24	742	
83	NT	OHE	NS	IC	Death	G3	36	1854	
84	NT	OHE	NS	P	LTFU	G2	N	332	
85	NT	OHE	NS	P	LTFU	G3	6	581	
86	LM	OHE		CE	LTFU	G4	2	324	
87	LM	OHE		CE	LTFU	G1	N	1107	
88	LM	OHE		CE	LTFU	G1	N	343	
89	BM	OHE		CE	LTFU	G4	3	587	
90	PM	OHE		CE	LTFU	G1	N	1842	
91	NT		NS	P	Death	G2	N	132	
92	PM	OHE		CE	LTFU	G2	N	412	
93	LM			CE	LTFU	G1	N	2143	
94	BM	OHE		CE	LTFU	G3	N	2081	
95	NT		NS	P	LTFU	U	N	37	

Abbreviations: NT, no treatment; N/A, not applicable; LM, lumpectomy; PM, partial mastectomy; UM, unilateral mastectomy; BM, bilateral mastectomy; OHE, ovarian hysterectomy; NS, NSAIDs; CE, complete excision; IC, incomplete excision; P, palliative treatment; N, no treatment; LTFU, lost to follow-up; G1–5, Grade 1–5; ST, survival time; DFI, disease-free interval, N, no recurrence.

**Table 4:** Survival parameters for dogs with mammary carcinomas, according to clinical stage (*p* = 0.025, n = 95).

Stage	Number of Dogs	Censored	Median (Days)	95% CI
1	28	8	1046	869–1314
2	16	5	853	590–1066
3	32	4	564	476–909
4	15	3	324	177–646
5	4	1	123	33–213

**Table 5:** Survival parameters for dogs with mammary carcinomas, according to medical treatments (*p* = 0.001, n = 95).

Treatment Type	Number of Dogs	Censored	Median (Days)	95% CI
No treatment	9	0	172	73–469
Palliative treatment	17	2	140	160–235
Incomplete excision	13	1	324	261–756
Complete excision	56	22	992	934–1217

**Table 6:** Comparison of clinical outcomes between incomplete and complete surgical treatments (*p* = 0.015, n = 76).

Stage	Number of Dogs	Censored	Median (Days)	95% CI
Complete excision				
1	23	11	1184	1090–1478
2	14	3	912	696–1144
3	15	4	1041	739–1367
4	4	2	607	252–860
Incomplete excision				
1	4	2	657	321–931
2	4	1	412	220–832
3	5	1	527	157–766
4	7	2	323	250–569

**Table 7:** Cox regression results for survival time across treatment groups, adjusted for age and OHE treatment.

Variable	Hazard Ratio		
	Mean	95% Confidence Interval	
		Lower	Upper
Surgical Type			
No treatment			
Lumpectomy	0.05	0.01	0.28
Partial mastectomy	0.02	0.002	0.10
Unilateral mastectomy	0.06	0.004	0.77
Bilateral mastectomy	0.004	0.0002	0.08
OHE			
None			
Treated	2.49	0.83	7.47
Age	1.34	1.12	1.60

## Data Availability

All data in this study are included in the article.
